# Association of PPAR Alpha Intron 7 G/C, PPAR Gamma 2 Pro12Ala, and C161T Polymorphisms with Serum Fetuin-A Concentrations

**DOI:** 10.1155/2017/7636019

**Published:** 2017-07-11

**Authors:** Bernadett Márkus, Krisztián Vörös, Dorina Supák, Zsolt Melczer, Károly Cseh, László Kalabay

**Affiliations:** ^1^Department of Family Medicine, Semmelweis University, Budapest, Hungary; ^2^2nd Department of Obstetrics and Gynecology, Semmelweis University, Budapest, Hungary; ^3^Institute of Public Health, Semmelweis University, Budapest, Hungary

## Abstract

**Background:**

Both peroxisome activator proteins (PPARs) and fetuin-A play a role in lipid and glucose metabolism.

**Aims:**

We investigated whether PPAR*α* intron 7 G2468/C and PPAR*γ*2 Pro12Ala and PPAR*γ* exon 6 C161T polymorphisms are associated with serum fetuin-A concentrations.

**Patients and Methods:**

The PPAR*α* intron 7 G/C polymorphism was studied in cohort 1 (79 reference individuals, 165 postinfarction patients). The two PPAR*γ* polymorphisms were investigated in cohort 2 (162 reference individuals, 165 postinfarction patients). Fetuin-A levels and PPAR polymorphisms were determined by radial immunodiffusion and polymerase chain reaction-restriction fragment length polymorphism techniques.

**Results:**

The C allele variant of PPAR*α* intron 7 G2467C was associated with higher fetuin-A levels (*p* = 0.018). Postinfarction status (*p* = 0.001), PPAR*α* intron 7 GG/GC/CC genotypes (*p* = 0.032), and the C allele (*p* = 0.021) were the strongest determinants of fetuin-A concentration in a multiple regression model. Higher fetuin-A levels were associated with the Pro variant of PPAR*γ*2 (*p* = 0.047). Postinfarction status (*p* = 0.041) and BMI (*p* < 0.001) but not PPAR*γ*2 Pro were the strongest determinants of fetuin-A concentrations. PPAR*γ* exon 6 C161T genotypes were not associated with fetuin-A levels.

**Conclusions:**

Fetuin-A was determined mainly by the PPAR*α* intron 7C allele and postinfarction status in cohort 1 and the BMI and postinfarction in cohort 2. The PPAR*α* intron 7C and PPAR*γ*2 Pro variants are associated with fetuin-A levels.

## 1. Introduction

Human fetuin-A is a multifunctional hepatic glycoprotein that has been involved in the development of obesity [1–3], insulin resistance [4], metabolic syndrome [1, 5], type 2 diabetes [6–8], adipocyte dysfunction [9], and fatty liver [4].

Peroxisome proliferator-activated receptors (PPARs) are members of the nuclear hormone receptor superfamily of ligand-activated transcription factors. The PPAR subgroups PPAR*α*, PPAR*β*/*δ*, and PPAR*γ* (*γ*1 and *γ*2) play an important role in the pathogenesis of these processes, which has been extensively reviewed [10–12].

There are several observations suggesting a relationship between serum fetuin-A levels and activities of different PPARs. For example, the direct inhibitory effect of pioglitazone on hepatic fetuin-A expression has been observed in rats [13] and in humans [14]; the former was reversed by GW9602, direct PPAR*γ* inhibitor.

Polymorphisms of PPAR*γ* and PPAR*α* have also been described and found to be associated with disorders of hyperlipidemia, glucose homeostasis, and diabetes. Thus the C allele of the PPAR*α* intron 7 polymorphism was found to be more frequent in patients with myocardial infarction and dyslipidemia [15]. The T allele of the PPAR*γ* exon 6 C161T polymorphism was supposed to have protective role against coronary artery disease in Chinese population [16], whereas others found that this allele was associated with an increased risk for coronary heart disease [12]. The association between polymorphic variants of PPAR and serum fetuin-A levels, however, has not been investigated yet.

In our study, we aimed to investigate whether polymorphisms of PPAR*γ* (PPAR*γ*2 Pro12Ala and exon 6 C161T) and PPAR*α* (intron 7 G2467C) are associated with or may affect serum fetuin-A levels in two cohorts.

## 2. Patients and Methods

Three-hundred and forty-two patients were originally involved in this study. Exclusion criteria were as follows: clinical or laboratory signs of acute vascular disease (myocardial infarction, stroke), acute infection, malignant tumor, hepatic disease, renal failure, immune suppression, severe medical or surgical conditions, and trauma. Finally, we had 327 patients (cohort 2) who had all comparable data (including successful genotyping for both PPAR*γ* polymorphisms). We were able to perform successful PPAR*α* genotyping in a smaller number of patients (cohort 1, *n* = 244). The genotyping success rate was greater than 99% in both cohorts. The genotypes were in the Hardy-Weinberg equilibrium.

PPAR*α* polymorphism was studied in cohort 1. This cohort comprised 244 individuals (120 men and 124 women, age: 60.1 ± 11.2 years, mean ± SD). Cohort 1 consisted of 79 reference individuals (15 men, 64 women, age: 61.0 ± 9.4 years) and 165 patients surviving myocardial infarction (105 men, 60 women, age: 59.6 ± 12.2 years).

PPAR*γ* polymorphisms were studied in cohort 2. This cohort consisted of 327 individuals (161 men, 166 women, age: 57.9 ± 13.0 years). This cohort consisted of 162 reference subjects (61 men, 101 women, age: 56.1 ± 13.8 years) and the same 165 postinfarction patients as in cohort 1 (105 men, 60 women, age: 59.6 ± 12.2 years).

Postinfarction patients had a history of STEMI myocardial infarction (6–24 months prior to the start of the study). Diabetes was diagnosed based on fasting plasma glucose > 7.0 mmol/l or the 2-hr OGTT > 11.1 mmol/l. Patients with diabetes were treated with diet, metformin, and bedtime insulin.

All persons gave their informed consent prior to their inclusion in the study. The study was approved by the local Ethics Committee of the Károlyi Sándor Municipality Hospital.

### 2.1. Determination of PPAR Polymorphic Variants

The determination of the PPAR*γ* and PPAR*α* variants was performed by PCR-RFLP technique.

The PPAR*α* G2467C intron 7 polymorphism (rs 4253778) was studied by PCR-RFLP technique, using a 5′ forward primer of ACA ATC ACT CCT TAA ATA TGG TGG and a 3′ reverse primer of AAG TAG GGA CAG ACA GGA CCA GTA. The PCR product was digested with Taq1 (New England Biolabs, Boston, MA, USA) resulting in one fragment of 266 bp of the carriers of the wild-type allele and two fragments of 216 and 50 bp in the carriers of the mutant allele (thermocycles 94°C 15 min, 30 × 94°C 30 sec, 50°C 20 sec, and 72°C 30 sec) [17].

For PPAR*γ* Pro12Ala (rs 1801282) polymorphism, we used a 5′ forward primer of GCC AAT TCA AGC CCA GTC and a mutagenic 3′ reverse primer of GAT ATG TTT GCA GAC AGT GTA TCA GTG AAG GAA TCG CTT TCC G. The PCR product was digested with Bst U1 enzyme (New England Biolabs, Boston, MA, USA) resulting in one fragment of 270 bp in the carriers of wild-type and two fragments of 227 and 43 bp in carriers of mutant allele (thermocycles 95°C 15 min, 35 × 94°C 30 sec, 65°C 45 sec, and 72°C 1 min) [18].

The exon 6 polymorphism C161T of PPAR*γ* (rs 3856806) was investigated by PCR-RFLP technique using a 5′ forward primer of CAA GAC AAC CTG CTA CAA GC and a 3′ reverse primer of TCC TTG TAG ATC TCC TGC AG. The PCR product was digested with Pml1 enzyme (New England Biolabs, Boston, MA, USA) resulting in two fragments of 120 and 80 bp in carriers of the wild-type allele and only one fragment of 200 bp in the carriers of the mutant allele (thermocycles 94°C 15 min, 30 × 94°C 30 sec, 56°C 30 sec, and 72°C 30 sec) [19].

### 2.2. Determination of Serum Fetuin-A Concentration

Serum fetuin-A concentrations were determined by radial immunodiffusion using the commercially available product (anti-fetuin-A, IgG fraction, Incstar, cat. number 81931, 13.7 mg/ml, in a final concentration of 84 *µ*l/11.5 ml gel), as previously described [20].

### 2.3. Determination of Insulin Resistance Parameters

Plasma glucose and insulin were determined by the routine HK-G6P-DH and ELCIA methods, respectively. The Homeostasis Model Assessment-Insulin Resistance (HOMA-IR) model was calculated according to Matthews et al. [21].

### 2.4. Statistical Analysis

Statistical analysis was carried out using the SPSS v.21 statistical software (SPSS Inc., Chicago, IL, USA). Nonparametric methods, including the Bonferroni (Dunn) post hoc test, were used. *p* values < 0.05 were considered as significant.

## 3. Results

### 3.1. Subject Characteristics

The characteristics of the study participants are shown in Table 1.

Sixty-five per cent of the postinfarction patients received statins and 70% of them aspirin. Serum fetuin-A concentrations did not differ statistically between patients treated and not treated with these two medications (687 ± 122 versus 636 ± 81 mg/l, *p* = 0.204 for statins and 665 ± 0.120 versus 672 ± 124 mg/l, *p* = 0.795 for aspirin, resp.).

### 3.2. PPAR*α* Intron 7 G/C, PPAR*γ*2 Pro12Ala, and PPAR*γ* Exon 6 C161T Allele Distribution in Postinfarction Patients and Reference Individuals

The distribution of PPAR*γ* and PPAR*α* alleles is shown in Table 2. PPAR*γ* Pro12Ala and PPAR*α* intron 7 G/C alleles did not differ significantly between postinfarction patients and reference subjects. Postinfarction patients, however, had a significantly higher T allele frequency of the PPAR*γ* C161T compared to reference subjects.

### 3.3. Analysis of Association between PPAR*α* Intron 7 G2467C Variants and Serum Fetuin-A Concentrations

Serum fetuin-A levels of individuals with the CC genotype were higher than those of GG genotype (Table 3). In the dominant model (C versus non-C nucleotide), individuals with the minor variant C allele had significantly higher serum fetuin-A concentrations than those with the non-C. In a recessive model (G versus non-G nucleotide), there was no difference between the two variants (651 ± 107 mg/l, *n* = 238 versus 662 ± 171 mg/l, *n* = 6, *p* = 0.702).

Except for age, serum fetuin-A concentrations showed significant correlations with parameters listed in Table 4. Serum fetuin-A levels associated weakly but significantly with PPAR*α* intron 7 GG/GC/CC genotypes and the C allele but not with the G allele. During partial correlation analysis, however, the correlation between fetuin-A concentrations and PPAR*α* intron 7 GG/GC/CC genotype lost significance when corrected for BMI (*r* = 0.100, *p* = 0.125), diabetes status (*r* = 0.108, *p* = 0.092), HOMA-IR (*r* = 0.116, *p* = 0.072), and postinfarction status (*r* = 0.122, *p* = 0.058). Correlation between fetuin-A levels and the PPAR*α* intron 7C allele also became insignificant when corrected for BMI (*r* = 0.107, *p* = 0.095), diabetes status (*r* = 0.115, *p* = 0.072), and HOMA-IR (*r* = 0.123, *p* = 0.056) but remained significant after correction for postinfarction status (*r* = 0.131, *p* = 0.041).

The results of the univariate linear regression analysis between independent variables (predictors) and serum fetuin-A concentration (dependent variable) are shown in Table 5. Serum fetuin-A levels showed weak but statistically significant data with all investigated potential predictors, including PPAR*α* intron 7 G/C genotypes and the C allele but not with age. Thus age was excluded from further analysis.

Next we investigated whether PPAR*α* intron 7 G/C genotype and the C allele may determine serum fetuin-A concentration in a multiple regression model (Table 6). In the model containing all independent parameters, we investigated the PPAR*α* intron 7 GG/GC/CC genotype and the C allele were the only statistically significant predictors of serum fetuin-A levels. In a backward stepwise regression model, the postinfarction status, the PPAR*α* intron 7 GG/GC/CC genotypes, and the C allele proved to be the strongest determinants of fetuin-A concentration.

### 3.4. Analysis of Association between PPAR*γ*2 Pro12Ala Variants and Serum Fetuin-A Concentrations

Patients with Pro/Pro and Pro/Ala genotype had significantly higher serum fetuin-A concentrations than those with the Ala/Ala genotype (Pro/Pro: 681 ± 131 mg/l, *n* = 247, Pro/Ala: 706 ± 131 mg/l, *n* = 75, and Ala/Ala: 565 ± 116 mg/l, *n* = 5, *p* = 0.043, Kruskal-Wallis test). In the recessive model (Pro versus non-Pro), the fetuin-A level associated with the allele determining Pro exceeded that of the non-Pro variant (687 ± 131 mg/l, *n* = 322 versus 565 ± 116 mg/l, *n* = 5, *p* = 0.047, Mann–Whitney test). Fetuin-A concentrations did not differ in the recessive model (Ala versus non-Ala) (698 ± 143 mg/l, *n* = 80 versus 681 ± 131 mg/l, *n* = 247, *p* = 0.287).

Serum fetuin-A concentration was significantly associated with BMI, HOMA-IR, gender, and the Pro allele but not with the diabetes and postinfarction status, PPAR*γ* Pro/Pro, Pro/Ala, and Ala/Ala genotypes or the Ala allele (Table 7). Thus these two latter parameters were left out from further analysis. The correlation between fetuin-A concentration and the Pro allele was lost following correction for BMI and gender but not with HOMA-IR (Table 8).

Univariate regression analysis showed that serum fetuin-A (dependent variable) weakly but significantly correlated with BMI and the PPAR*γ* Pro allele (independent variable, Table 9). This latter independent variable lost its predictor role when BMI was included in the regression model.

In the multiple backward stepwise regression model, postinfarction status (*β* = 0.111, *p* = 0.041) and BMI (*β* = 0.426, *p* < 0.001) proved to be the strongest determinants of fetuin-A concentrations.

### 3.5. Analysis of Association between PPAR*γ* Exon 6 C161T Variants and Serum Fetuin-A Concentrations

We found no significant differences among serum fetuin-A concentrations of individuals with different PPAR*γ* exon 6 C161T genotypes, nor between the C and non-C or T and non-T groups (Table 10). Fetuin-A levels did not correlate with the PPAR*γ* C161T genotypes, C, and T alleles, either (data not shown).

There were 3 minor variant homozygotes (TT) in the postinfarction but none in the reference group. Thus the genotype distribution of postinfarction patients markedly differed from that of reference individuals (CC/CT/TT: 130/37/3 versus 140/17/0, *p* = 0.006). The T allele was significantly more frequent among postinfarction patients compared to reference group (40/170 = 23.5% versus 17/157 = 10.8%, *p* = 0.002). Accordingly, postinfarction patients with the CC genotype had lower fetuin-A levels than reference subjects (668 ± 113 mg/l, *n* = 130 versus 710 ± 146 mg/l, *n* = 140, *p* = 0.037).

## 4. Discussion

In our study, we investigated whether PPAR*α* intron 7 G/C, PPAR*γ*2 Pro12Ala, and PPAR*γ* C161T variants are associated with serum fetuin-A concentration. Since subjects in our groups had several parameters that are known to affect fetuin-A levels such as age, gender, BMI, parameters of insulin resistance (diabetes status, HOMA-IR), and postinfarction status [22], we chose regression model to estimate the impact of these variables.

PPAR*α* has been termed as a lipid sensor and is involved in microsomal *ω*-oxidation and mitochondrial and peroxisomal *β*-oxidation resulting in energy burning and reduced fat storage [11]. The minor variant of the PPAR*α* intron 7C has been considered to have a decreased activity compared to G, the major variant. The C haplotype promotes the early development of type 2 diabetes [17] and is more frequent among postinfarction patients [12, 15]. Doney et al. have found that the risk of myocardial infarction is higher in the presence of the C allele [23]. We also have found that the C allele is associated with higher fetuin-A levels and the multiple regression revealed that fetuin-A levels are strongly determined by the postinfarction status and remarkably by the PPAR*α* intron 7 G/C polymorphism, as well. Although the C allele was not more frequent among our postinfarction patients, the higher fetuin-A concentration may also have deleterious effects in them. Elevated fetuin-A concentration is a marker of fatty liver, characterized by decreased *β*-oxidation of fatty acids [4].

Fetuin-A is synthesized almost exclusively by the hepatocytes in adults [24] and the PPAR*α* is mainly expressed in the liver, as well. Fetuin-A is known as an endogenous ligand that binds to free fatty acids and functions as an endogenous ligand for the Toll-like receptor TLR-4 thereby linking metabolic diseases (hyperlipidemia, insulin resistance) and subclinical inflammation [25]. This “missing link” character of fetuin-A has been further supported by the clinical studies of Stefan and Häring [26]. These findings are in line with our observation that postinfarction status, an endpoint of subclinical inflammation, and PPAR*α* intron 7C allele were the strongest determinants of fetuin-A in our subjects.

Compared to Ala metabolically disadvantageous characteristics are attributed to the Pro variant of PPAR*γ*2 Pro12Ala [27]. Indeed, we found only the Pro allele among individuals with BMI over 25 kg/m^2^ and only lean (BMI ≤ 25 kg/m^2^) subjects had the Ala/Ala homozygous variant. Nevertheless, even among individuals with the Ala variant, obesity was associated with higher fetuin-A levels compared to lean ones. Patients with diabetes had higher fetuin-A levels, the difference being significant in Pro/Pro major allele homozygotes (704 ± 124 mg/l, *n* = 64 versus 673 ± 132 mg/l, *n* = 183, *p* = 0.020). Since fetuin-A is known to be the natural inhibitor of the insulin receptor tyrosine kinase, the Pro allele may convey increased insulin resistance. Indeed, we found minor variant Ala to be more frequent among nondiabetics (68/251 = 27.1%) compared to diabetics (12/76 = 15.8%, *p* = 0.044). This finding is in accordance with that of Vergotine, who observed that the Pro allele increases insulin resistance, along with IRS1Gly972 [28]. Conversely, the minor allele Ala seemed to be protective in Iranian and Chinese populations [29, 30]. In our model, however, the relationship of fetuin-A levels with BMI and postinfarction status was much stronger than the one with insulin resistance (diabetes status or HOMA). The Pro allele was associated with higher fetuin-A in the nondiabetic group, as well, which is reflected by the weak correlation with diabetes status and HOMA-IR. Given the property of fetuin-A to increase insulin resistance, its weak association with the Pro allele can contribute to the deleterious effects of this allele.

Analysis of the effect of the PPAR*γ* C161T variants on serum fetuin-A concentrations yielded the least consistent results. We had 3 minor homozygotes (TT) among the postinfarction patients but not among the reference subjects. This finding is in accord with the observation of Qian et al., who found that coronary heart disease was not associated with the T-carrier state but these individuals had higher risk for acute coronary heart syndrome [12]. Wu et al. found mild protective effect of the T allele only in the Chinese but not in other populations [16]. We found no marked associations of C161T genotypes and alleles neither in postinfarction patients nor in the reference group, and not during the analysis of nonobese, nondiabetic individuals. This suggests that the C161T has the weakest association with fetuin-A levels out of the three PPAR polymorphisms we studied. Since PPAR*γ* is expressed mainly in the fat tissue, its association with the levels of the liver secretory protein fetuin-A cannot be as close as that of PPAR*α*.

Although not yet entirely clarified, several observations suggest the molecular basis of the association between PPAR variants and fetuin-A synthesis. The PPAR*α* agonist fibrates decrease fetuin-A expression in obese patients with or without type 2 diabetes mellitus [31]. The PPAR*γ* agonist pioglitazone strongly inhibits fetuin-A expression [14]. The upregulation of both PPAR*α* and PPAR*γ* results in the downregulation of fetuin-A and NF*κ*B and upregulation of the AMPK kinase activities. Palmitate, the oxidation of which is highly induced by PPAR*α*, has been shown to stimulate NF*κ*B binding to the fetuin-A promoter [9]. Thus a less functional variant of PPAR*α* could finally result in enhanced fetuin-A expression.

Our study has its limitations. First, the sample size is not big enough to allow for analysis of a comparable number of minor variants. Second, our cohorts were not controlled for environmental factors and prescribed medication and dietary saturated and polyunsaturated fat as it has been suggested [32].

## 5. Conclusion

In summary, our results indicate a relatively close relationship between PPAR*α* intron 7 G/C and PPAR*γ*2 Pro12Ala variants and serum fetuin-A concentrations reflecting higher levels in the presence of the C allele of the former and the Pro allele of the latter one. It is very likely that these associations are obscured by obesity and/or diabetes. Larger scale studies are needed to further determine the biological and clinical significance of the PPAR polymorphisms on fetuin-A levels.

## Figures and Tables

**Table 1:** Subject characteristics.

	Cohort 1 (*n* = 244)		Cohort 2 (*n* = 327)	
	Reference individuals (*n* = 79)	Postinfarction patients (*n* = 165)	Reference individuals (*n* = 162)	Postinfarction patients (*n* = 165)
Gender (male/female)	15/64	105/60	61/101	105/60
Age (years, mean ± SD)	61.0 ± 9.4	59.6 ± 12.2	56.1 ± 13.8	59.6 ± 12.2
BMI (kg/m^2^)	24.1 ± 1.6	28.1 ± 4.2^*∗∗*^	27.4 ± 0.4	28.1 ± 4.2^*∗*^
Obesity (no/yes)	68/11	46/119^*∗∗*^	78/84	46/119^*∗∗*^
Diabetes status (no/yes)	79/0	112/53^*∗∗*^	139/23	112/53^*∗∗*^
HOMA-IR	1.0 ± 0.2	6.2 ± 4.6^*∗∗*^	1.5 ± 1.5	6.1 ± 4.6^*∗∗*^

^*∗*^ *p* < 0.01 and ^*∗∗*^*p* < 0.001, compared to reference individuals; BMI: body mass index; HOMA-IR: Homeostasis Model Assessment-Insulin Resistance; Mann–Whitney test.

**Table 2:** The PPAR*α* intron 7 G/C, PPAR*γ*2 Pro12Ala, and PPAR*γ* exon 6 C161T allele distribution among postinfarction patients and reference individuals.

	Allelic frequency	*χ* ^2^	RR (95% CI)	OR (95% CI)	*p*
*PPARα intron 7 G/C (rs4253778)*						
G	P: 0.8273 R: 0.8165	0.0861	1.013 (0.9271–1.107)	1.077 (0.6571–1.764)	0.769
C	P: 0.1727 R: 0.1835				
*PPARγ2 Pro12Ala (rs1801282)*						
Pro12	P: 0.8618 R: 0.8789	0.4279	0.9804 (0.9241–1.040)	0.8583 (0.5428–1.3570)	0.513
Ala12	P: 0.1382 R: 0.1211				
*PPARγ exon 6 C161T (rs3856806)*						
C	P: 0.8735 R: 0.9459	10.25	0.9235 (0.8799–0.9693)	0.3953 (0.2204–0.7091)	0.001
T	P: 0.1265 R: 0.0541				

P: postinfarction patients; R: reference subjects; *p*: *χ*^2^ test.

**Table:** (a) PPAR*α* intron 7 G2467C polymorphisms

GG		GC		CC		*p*
Fetuin-A mg/l	*n*	Fetuin-A mg/l	*n*	Fetuin-A mg/l	*n*	
641 ± 150	164	671 ± 110	74	662 ± 170	6	0.040^#^

**Table:** (b) PPAR*α* intron 7 G2467C alleles

C allele		non-C		*p*
Fetuin-A mg/l	*n*	Fetuin-A mg/l	*n*	
670 ± 114	80	641 ± 105	164	0.018^§^

^#^Kruskal-Wallis test; ^§^Mann–Whitney test.

**Table 4:** Correlation between serum fetuin-A and investigated parameters (*n* = 244).

Parameter	Correlation coefficient	*p*
BMI	0.167	0.009
Diabetes status (no/yes)	0.133	0.038
HOMA-IR	0.205	0.001
Age	−0.109	0.188
Gender	−0.168	0.017
Postinfarction status (no/yes)	0.277	<0.001
PPAR*α* GG/GC/CC	0.144	0.025
PPAR*α* C allele	0.151	0.018
PPAR*α* G allele	0.025	0.700

BMI: body mass index; HOMA-IR: Homeostasis Model Assessment-Insulin Resistance; Spearman correlation.

**Table 5:** Univariate regression analysis between serum fetuin-A concentrations and metabolic parameters (*n* = 244).

Predictor	Standardized *β*	*p*
BMI	0.146	0.023
Diabetes status (no/yes)	0.136	0.034
HOMA-IR	0.163	0.011
Postinfarction status (no/yes)	0.212	0.001
Age	−0.137	0.098
Gender	−0.149	0.027
PPAR*α* GG/GC/CC	0.131	0.042
PPAR*α* C allele	0.140	0.029

BMI: body mass index; HOMA-IR: Homeostasis Model Assessment-Insulin Resistance.

**Table 6:** Multiple regression analysis of PPAR*α* intron 7 G/C genotypes and C allele and serum fetuin-A concentration (*n* = 244).

Predictor	PPAR*α* intron 7 GG/GC/GG		PPAR*α* intron 7 C allele	
	Standardized *β*	*p*	Standardized *β*	*p*
All predictors included				

BMI	0.027	0.708	−0.024	0.743
Diabetes status (no/yes)	0.054	0.474	0.045	0.551
HOMA-IR	0.037	0.651	0.035	0.666
Postinfarction status (no/yes)	0.130	0.127	0.132	0.121
Gender	−0.079	0.260	−0.083	0.242
PPAR*α* intron 7 genetics^#^	0.134	0.036	0.144	0.024
	Model fit: *p* = 0.006		Model fit: *p* = 0.005	

Stepwise backward regression				

Postinfarction status (no/yes)	0.214	0.001	0.215	0.001
PPAR*α* intron 7 genetics^#^	0.135	0.032	0.144	0.021
	Model fit: *p* < 0.001		Model fit: *p* < 0.001	

^#^Regression parameters of the PPAR*α* intron 7 GG/GC/GG genotypes are indicated in the second and the third and those of the PPAR*α* intron 7 C allele in the fourth and fifth columns of the table, respectively; BMI: body mass index; HOMA-IR: Homeostasis Model Assessment-Insulin Resistance.

**Table 7:** Correlation between serum fetuin-A and investigated parameters (*n* = 327).

Parameter	Correlation coefficient	*p*
BMI	0.426	<0.001
Diabetes status (no/yes)	0.102	0.064
HOMA-IR	0.129	0.027
Gender	−0.178	0.008
Postinfarction status (no/yes)	0.098	0.078
PPAR*γ* Pro/Pro, Pro/Ala, and Ala/Ala	0.050	0.365
PPAR*γ* Pro allele	0.130	0.018
PPAR*γ* Ala allele	0.052	0.349

BMI: body mass index HOMA-IR: Homeostasis Model Assessment-Insulin Resistance; Spearman correlation.

**Table 8:** Partial correlation between serum fetuin-A concentrations and PPAR*γ* Pro allele (*n* = 327).

Corrected for	Correlation coefficient	*p*
Uncorrected	0.130	0.018
BMI	0.069	0.215
HOMA-IR	0.129	0.027
Gender	0.129	0.055

**Table 9:** Univariate regression analysis between serum fetuin-A concentrations and predictor parameters (*n* = 327).

Predictor	Standardized *β*	*p*
BMI	0.520	<0.001

Diabetes status (no/yes)	0.065	0.239

HOMA-IR	0.059	0.291

Postinfarction status (no/yes)	0.137	0.013

PPAR*γ* Pro allele	0.127	0.022

PPAR*γ* Pro allele + BMI	0.059 0.512	0.215 <0.001

BMI: body mass index; HOMA-IR: Homeostasis Model Assessment-Insulin Resistance.

**Table:** (a) PPAR exon 6 C161T polymorphisms

CC		CT		TT		*p*
Fetuin-A mg/l	*n*	Fetuin-A mg/l	*n*	Fetuin-A mg/l	*n*	
690 ± 133	270	661 ± 125	54	641 ± 104	3	0.340^#^

**Table:** (b) PPAR*γ* exon 6 C161T Ala Allele

Fetuin-A mg/l	*n*	Fetuin-A mg/l	*n*	*p*
C		non-C		
685 ± 132	324	641 ± 104	3	0.674^§^

T		non-T		
659 ± 123	57	690 ± 133	270	0.138^§^

^#^Kruskal-Wallis test; ^§^Mann–Whitney test.
